# The 18-month efficacy of an Intensive LifeStyle Modification Program (ILSM) to reduce type 2 diabetes risk among rural women: a cluster randomized controlled trial

**DOI:** 10.1186/s12992-023-00910-3

**Published:** 2023-01-26

**Authors:** Qinyi Zhong, Yao Chen, Mengchun Luo, Qian Lin, Jianghong Tan, Shuiyuan Xiao, James Allen Willey, Jyu-Lin Chen, Robin Whittemore, Jia Guo

**Affiliations:** 1 Xiangya School of Nursing, Central South University, 172 Tongzipo Road, Changsha, 410013 Hunan China; 2 Manchester Centre for Health Psychology, School of Health Science, University of Manchester, Manchester, Greater Manchester M13 9PL UK; 3 Maternal and Child Health Hospital of Yongding, Zhangjiajie, Hunan 427000 People’s Republic of China; 4 Xiangya School of Public Health, Central South University, Changsha, Hunan 410013 People’s Republic of China; 5 Zhuzhou Central Hospital, Zhuzhou, Hunan 412000 People’s Republic of China; 6 Department of Family and Community Medicine, University of California, San Francisco, CA 94118 USA; 7 School of Nursing, University of California, San Francisco, CA 94118 USA; 8 School of Nursing, Yale University, New Haven, CT 06520 USA

**Keywords:** Woman, Gestational diabetes, Less-developed area, Prevention, Type 2 diabetes mellitus, Randomized controlled trial

## Abstract

**Background:**

Many lifestyle interventions have demonstrated efficacy up to one-year follow-up, yet maintaining improvements at longer-term follow-up is a well-recognized worldwide challenge, especially in underserved areas. The purpose of this study is to compare the 18-month efficacy of an Intensive LifeStyle Modification Program to usual care in reducing the risk for type 2 diabetes (T2D) among women with a history of gestational diabetes mellitus (GDM).

**Methods:**

We conducted a two-arm, cluster randomized controlled trial among women with a history of GDM in China. A total of 16 towns (clusters) in two distinct rural areas in south-central China were randomly selected (8 towns per area) and assigned (1:1) to the intervention (Intensive LifeStyle Modification Program) or control (usual care) group with stratification in the two rural areas. The strategies for maintaining intervention effects were used (including setting recursive goals and providing a supportive environment, etc.) under the guidance of social cognitive theory. The primary outcome was a change in T2D risk; secondary outcomes included glycemic, weight-related, behavioral, and psychological variables. All outcomes were collected at baseline, 6, and 18 months. All participants entered the intention-to-treat analysis. Data were analyzed via generalized estimation equation models (accounting for clusters) at the individual level, with subgroup analysis included in the model.

**Results:**

The sample included 320 women from 16 clusters (20 women per cluster). At 18 months, the intervention group demonstrated a significant improvement in T2D risk score, fasting blood glucose, body mass index (BMI), waist circumference, intention to eat low glycemic index food, perceived stress, quality of life in psychological and environmental domains, and social support over time (*p* < 0.05) based on the intention-to-treat analysis set. Subgroup analysis showed a significant interaction effect on T2D risk score in subgroups of different BMI, waist circumference, and blood glucose (*p* < 0.05).

**Conclusions:**

Over 18 months, the Intensive LifeStyle Modification Program reduced T2D risk among rural women with a history of GDM in China. Women who were overweight, had high abdominal adiposity, or had blood glucose intolerance benefited more from this intervention. This program serves as a potential diabetes prevention model for women with a history of GDM in low-resource settings worldwide.

**Trial registration:**

Registered on Chinese Clinical Trial Registry (ChiCTR1800015023) on 1st March 2018, http://www.chictr.org.cn/showproj.aspx?proj=25569

**Supplementary Information:**

The online version contains supplementary material available at 10.1186/s12992-023-00910-3.

## Introduction

Type 2 diabetes (T2D) affects approximately 425 million people worldwide and is projected to rise to 629 million by 2045, posing major health and economic consequences [1]. Gestational diabetes mellitus (GDM) history, as an independent T2D risk factor, confers a seven-fold risk of developing T2D, with the prevalence of GDM continuously increasing globally [2]. International Diabetes Federation (IDF) recommends lifestyle modification for individuals at elevated T2D risk because of the low cost and scalability without side effects. Many large-scale lifestyle trials around the world, including Finland, Australia, India, and China, etc., have successfully reduced the onset of diabetes among high-risk people and/or their risk factors, such as overweight, or obesity [3]. For women with a history of GDM, several large landmark clinical trials such as the Diabetes Prevention Program Outcomes Study also have shown that lifestyle interventions can effectively prevent or delay the onset of T2D [4].

However, maintaining lifestyle change over time is a well-recognized worldwide challenge, and few studies investigated the sustainability of lifestyle change in response to interventions [5, 6]. In a recent umbrella review, the maximum benefit of lifestyle interventions for weight management was typically achieved at 6-month with a plateau or relapse reported over time, which suggests that lifestyle intervention effects may diminish over time [7]. In addition, according to a recent systematic review, over 90% of existing lifestyle interventions were conducted within 12-month follow-ups [8]. Current evidence to support the longer-term (> 12 months) efficacy of lifestyle interventions is limited.

In China, 15.5% of the total population is at high risk of diabetes with the incidence of GDM reached 14.8% in 2020 [9–11]. Among them, the vast majority live in rural areas and are underserved due to socioeconomic disadvantages [12], which is not only reflected on limited access to diabetes prevention services, but also deriving less benefit from these services than urban population [13]. It was estimated that the medical expenditures of urban residents for diabetes prevention and treatment are 2.97 times that of rural residents, with about 16 healthcare providers per thousand in urban areas compared to six per thousand in rural areas in China [14], which is often the case in other developing areas such as Mexico, India, and Africa [15]. In addition, due to lower level of health literacy [16], rural residents tend to have difficulties in adopting and maintaining recommended behavior changes after lifestyle interventions, which may explain the rural-urban inequality of obtained benefit from lifestyle interventions [17]. Therefore, more sustainable diabetes prevention programs tailoring underserved high-risk groups are urgently needed.

Before evidence-based interventions are implemented into practice, it is necessary to consider modifying the intervention for subgroups of the population [18]. In a recent meta-analysis, lifestyle interventions were more effective in women with both glycemic intolerance and a history of GDM [19]. Yet, the relative magnitude of the lifestyle intervention efficacy in specific subgroups (e.g., BMI and ethnicity) has not been reported. Our research team contextually tailored the Intensive LifeStyle Modification Program (ILSM) for women with a history of GDM in low-resource rural areas of China. It was guided by the Social Cognitive Theory with active ingredients included promoting behavior initiation and maintenance [20]. The detailed description of the intervention is described in the methods section. Our preliminary 6-month efficacy of the ILSM showed significant improvement in glycemic status, waist circumference, and quality of life (*p* < .001) [21, 22]. Further subgroup analysis is needed to identify the impact of ILSM to specific high-risk populations along with.

determining its longer-term effects of ILSM on sustained T2D risk reduction among an underserved population.

The purpose of this study was to evaluate the 18-month efficacy of ILSM for women with a history of GDM on T2D risk score (as primary outcome), physiological (including glycemic and weight-related outcomes), and behavioral and psychosocial outcomes compared to the control group. We also conducted subgroup analyses, comparing women with different BMI, waist circumference, and blood glucose to determine the differential benefits of the ILSM.

## Methods

### Study design

This study utilized a cluster randomized controlled trial design to investigate the 18-month health impact of ILSM among 320 women with a history of GDM in rural China. The study was approved by the local ethics committee and registered at the Chinese Clinical Trial Registry. The details of the rationale, study and results of the 6-month follow-up have been published elsewhere [22, 23].

### Setting and randomization

The study was conducted in Yongding County and You County, Hunan Province in south-central China. Yongding County comprising 17 towns, has a large ethnic minority population (~ 50%, mainly Tujia and Miao population) in western Hunan Province; and You County comprising 14 towns, has a large ethnic Han population in eastern Hunan Province. The diversity (i.e., application to different geographic locations, ethnic groups, health care systems) of the included populations was used to test whether the ILSM program could be generalized to diverse underserved populations.

The town (cluster) was the unit of randomization, and a randomization protocol available on the internet (http://stattrek.com/statistics/random-number-generator.aspx) was used. No specific eligibility criteria for towns were identified. Eight towns were randomly selected from each county, yielding 16 towns. Following recruitment, the 16 towns were randomly allocated in a 1:1 ratio to either the intervention or the control group with stratification for the two counties, and each group included 8 towns (4 towns per county). Considering the realistic resource restriction in these towns (e.g., lack of resources in town-level hospitals, shortage of health care providers), two local county-level hospitals with the highest number of pregnant and birth-giving women (one per county), located in the center of the counties within a close distance from these towns, was chosen as the research sites to implement the study.

### Participants

Inclusion criteria were: 1) adult women with a history of GDM; 2) 6 weeks to 10 years postpartum; 3) living in the allocated towns and intending to live in these towns for at least 3 years; 4) having telephone access, and 5) able to read and speak in Mandarin Chinese. Exclusion criteria were: 1) women who were pregnant or planned to be pregnant within the next 3 years; 2) a diagnosis of diabetes; and 3) other serious health problems, such as physical or cognitive disability.

Eligible women who delivered babies within the past 10 years at the two research sites were identified through a medical record review. Local registered nurses who received standardized research protocol training contacted potential participants via telephone or in-person at a clinical visit. Nurses explained the research study to interested women, confirmed their eligibility, and obtained consent.

### Interventions

The timeline of the intervention activities and measurements for ILSM group and control group is showed in Table 1. Both groups received usual care based on current clinical guidelines, including general education about their T2D risk as well as a T2D prevention education brochure provided to each participant.

**Table 1:** The flowchart of the intervention activities and measurements

Time	Intensive LifeStyle Modification (ILSM) Group		Control Group	Measurements	
	Group seminar (Each 90 mins)	Telephone consultation (Each 20 mins)	Usual care	Outcomes	Measurement point
Baseline	I-1: orientation and goal-setting	□	A individual session to inform their T2D risk, and a brochure of general T2D prevention education was provided to each participants in the control group at baseline.	(1) the T2D risk score; (2) glycemic outcomes: FBG, 2 h-OGTT; (3)weight-related outcomes: BMI, waist circumference; (4) behavior outcomes: physical activity, fruit/vegetable consumption, intention to eat low glycemic-index foods; (5) psychological outcomes: perceived stress, quality of life, general self-efficacy, social support.	✓
At 1-Week	□	I-2: review I-1 and prepare for I-3			□
At 2-Week	I-3: healthy eating patterns	□			□
At 3-Week	□	I-4: review I-3 and prepare for I-5			□
At 4-Week	I-5: physical activity	□			□
At 5-Week	□	I-6: review I-5 and prepare for I-7			□
At 6-Week	I-7: stress management	□			□
At 7-Week	□	I-8: review I-7 and prepare for I-9			□
At 8-Week	I-9: family support & lifestyle patterns	□			□
At 9-Week	□	I-10: review I-9 and prepare for I-11			□
At 10-Week	I-11: relapse prevention and farewell	□			□
At 4-Month	□	I-12: encourage to stick to healthy lifestyle			□
At 5-Month	□	I-13: encourage to stick to healthy lifestyle			□
At 6-Month	□	I-14: encourage to stick to healthy lifestyle			✓
At 18-Month	□	□			✓

The intervention group also received the ILSM program from eight trained local nurses following the ILSM protocol reported elsewhere [23]. Before the ILSM program, the nurses received a structured five-day training from the research team using the Train the Trainer Model, which engages master trainers (research team members) in coaching new trainers (eight local nurses) to be competent to carry out the ILSM intervention. The ILSM training entailed self-study, class sessions, and live practice, with homework and practice between training. The nurses were required to pass a final evaluation held by our research team, which included a scenario simulation test and a personal interview to ensure they were equipped with essential intervention delivery skills.

Each nurse conducted the intervention for a group of ~ 20 participants from the same town at the research-designated hospitals. Participants were invited to attend six biweekly group seminars (90 minutes) and eight telephone consultation sessions (20 minutes each). The topics of group seminars included orientation and goal-setting, healthy eating patterns, physical activity, stress management, family support, family lifestyle patterns, and relapse prevention and farewell [23]. All content in the ILSM program was tailored to the context of rural women with a history of GDM. During the intervention period, a research assistant acted as a resource person at each research site and attended all sessions to assess intervention fidelity via a checklist (including evaluation concerning four domains: adherence, exposure, quality of delivery, and participant responsiveness). More details about the fidelity checklist are provided in Additional file 1: Appendix I.

To promote the long-term efficacy of the intervention, a series of strategies such as setting recursion goals and providing a environmental support, was used. For example, participants were required to set various small and easy-to-implement goals concerning health behavior at the early stage of intervention to ensure their early behavioral wins. These early wins help convince participants that behavior change is possible, thus induced into a recursive process and eventually sparking a positive and continual behavior change loop. In addition, participants were equipped with resources of health behavior change during the intervention, and these resources create supportive physical and psychological environment after intervention. By interacting with these essential elements and repeatedly reinforcing the desired behavior via environment, long-term benefits will tend to persist and reinforce the behavior change.

Due to the nature of educational and behavioral interventions, it was not feasible to blind participants and local nurses (investigators), though data assessors were blinded. Nurses and study participants were asked to sign an agreement that they would not share the intervention materials or protocol with others before the completion of the study.

### Outcome measures

Data were collected at baseline, 6-month, and 18-month. At each visit, all participants were invited to complete questionnaires on T2D risk assessment, lifestyle behavior, and psychological outcomes. At baseline, all participants also completed a demographic and clinical questionnaire. Data were collected in a quiet room at the research site by trained research assistants. At the same time, local nurses collected blood samples and completed physical examinations for physiological data. Data for women in intervention and control groups were collected on separate days to avoid contamination.

*The demographic and clinical data* included age, ethnicity, education, occupation, family income, and the number of months after delivery.

*Primary outcome:* T2D risk score was the primary outcome, as our target population was high T2D risk groups with normal glycemia rather than populations with impaired glucose tolerance. Adults with a high risk for T2D and normal glycemia have limited room to improve glycemic outcomes; thus, glycemic outcomes are unsuitable as a primary outcome. The T2D risk score was developed based on specific modifiable diabetes risk factors (such as BMI, waist circumference, physical activity, and dietary intake), and also includes some unmodifiable risk factors, which is in line with the theoretical mechanism of most diabetes prevention programs [24].

The T2D risk score was measured by the Chinese Diabetes Risk Scale (CHINARISK) [25], adapted from the Canadian Diabetes Risk Questionnaire [26]. This scale systematically combines modifiable and unmodifiable diabetes risk factors in order to identify people who may develop T2D in the next 10 years. Total scores range from 0 to 88; higher scores represent a greater risk of T2D. The questionnaire has a positive predictive value of 57% and a negative predictive value of 78%, with a sensitivity of 73% [25].

*Secondary outcome:* Glycemic outcomes included fasting blood glucose (FBG) and 2 h Oral Glucose Tolerance Test (OGTT) [27]. Venous blood samples were collected after overnight fasting, followed by blood samples taken 2 hours after consuming 75 g of glucose.

Weight-related outcomes included BMI and waist circumference. BMI was calculated by dividing body weight (kilograms) by height squared (meters); Waist circumference was measured at the midpoint between the highest point of the iliac crest and the lowest rib according to WHO standard [28].

Behavioral outcomes: Physical activity was assessed by the validated Chinese version of the International Physical Activity Questionnaire (Short Form) [29]; Fruit/vegetable consumption was measured by an item on the CHINARISK scale; The Intention to Eat Low Glycemic-index Foods was assessed with a 24-item questionnaire that uses a 7-point Likert scale, on which higher scores indicate a greater intention to eat low glycemic-index foods [30].

Psychological outcomes: Perceived stress was measured by the 14-item perceived stress scale [31]. Quality of life was assessed using the WHOQOL-BREF questionnaire, which includes 26 items and evaluates physiological, psychological, social relations, and environmental domains of quality of life. General self-efficacy was measured using the 10-item general self-efficacy questionnaires [32]. Social support was measured using the 10-item social support rating scale (SSRS) [33]. All the measurements used in this study with well-documented psychometric properties and have been used in Chinese populations in China [34–37].

### Statistical analysis

The analyses were done at the individual level in SPSS (Version 22.0; Armonk, NY, United States). The double-entry data method was adopted to ensure data accuracy via the EpiData 3.0 software (EpiData Association, Odense, Denmark). All randomly assigned participants (*N* = 320) entered the intention-to-treat analysis, which means that the randomization groups were used in this analysis, irrespective of any protocol violations. We ensured that the outcomes for participants who withdrew from the trial prior to the 6- or 18-month were retained in the analysis.

The data were presented as means with SDs or as counts with percentages. Descriptive statistics were used to describe demographic and clinical characteristics. The demographic and clinical data of the intervention and control groups were compared using two independent samples t-tests and Chi-square tests. Repeated measure analyses (three points) were conducted using a generalized estimating equation (GEE) model to compare the differences between two groups in T2D risk scores, glycemic, weight-related, behavioral, and psychological variables from baseline to 18-month follow-up. GEEs were developed as an extension of the general linear model to analyze longitudinal and other correlated data. GEE models take into account the correlation between repeated measurements in the same subject; models do not require complete data and can be fitted even when individuals do not have observations at all time points [38]. We added an interaction term (group by time) to each model to investigate the interactive effects of intervention and time. Models were performed unadjusted and adjusted for age, months after delivery, ethnicity, education, marriage, occupation, and family income. To assess the potential effects of clustering, we calculated the eta^2^ coefficient for each of the three outcomes and three time periods. The eta^2^ values for FBG ranged from 0.319 to 0.491. Coefficients for OGTT-2 h and the T2D risk score were substantially smaller, ranging from 0.049 to 0.114. In general, larger eta^2^ values are associated with larger standard errors, wider confidence intervals, and more conservative *p*-values. In order to adjust for such effects, we used fixed effects GEE regression models where the cluster itself is included as a factor within the model.

To assess if the intervention effect was statistically different between subgroups, we conducted several subgroup analyses for T2D risk scores: research site (You County, Yongding County), ethnicity (Han, minority), BMI (≤24 kg/ m2, > 24 kg/ m2), waist circumference (≤80 cm, > 80 cm), glucose dysregulation (FBG ≤6.1 mmol/L or OGTT-2 h ≤7.8 mmol/L, FBG > 6.1 mmol/L or OGTT-2 h > 7.8 mmol/L) and months after delivery (≤12, > 12). We included interaction terms (group by subgroup) in the GEE models to assess differences between subgroups.

## Results

### Baseline characteristics and participant retention

**Fig. 1:**
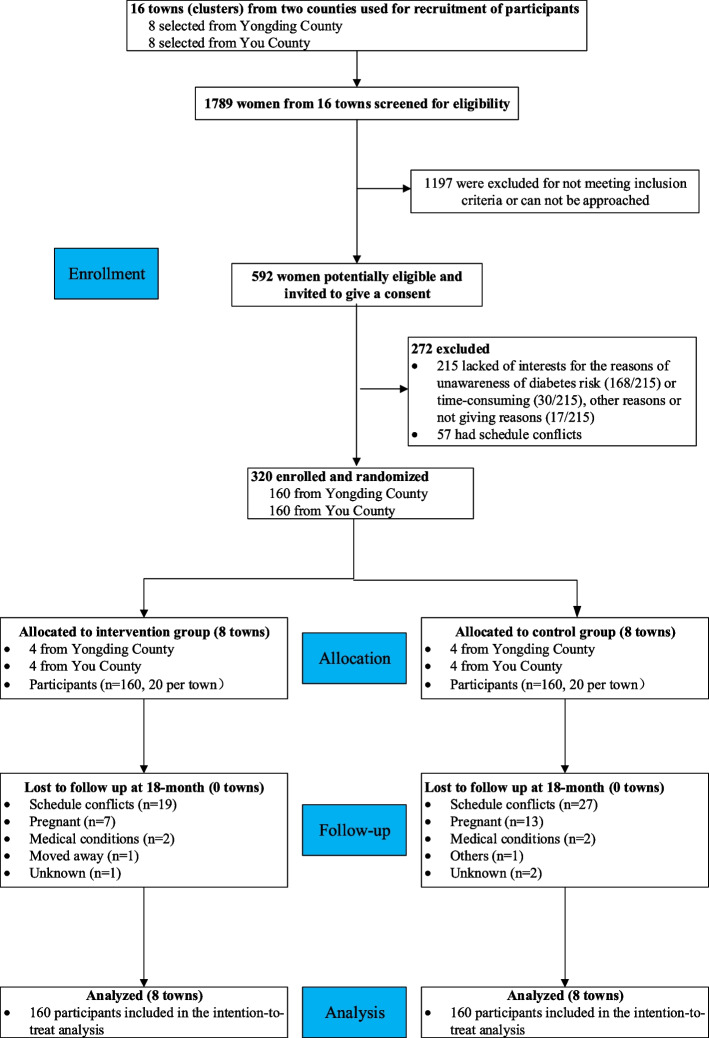
CONSORT flow diagram

Eight towns were selected from each county, yielding a total of 16 towns for this study. Recruitment included a screening of 1789 individuals from these towns, of which 757 (42.3%) were excluded due to not meeting the inclusion criteria, 440 (24.6%) could not be reached; the remaining 592 (33.1%) were eligible for further assessment. Of these, 272 (45.95%) people declined participation due to lack of interest (215, 36.3%) or schedule difficulties (57, 9.6%), and 320 with an average of 20 women per town enrolled. Followed by the intention-to-treat principle, data analyses included all participants randomly assigned to the intervention group (8 towns, 160 participants) or control group (8 towns, 160 participants), no matter whether they completed follow-up measurements or received assigned interventions. Participant flow is presented in the CONSORT diagram (Fig. 1).

In the overall intention-to-treat sample, the mean age (± SD) was 31.92 (± 4.91) years. About 55.3% were of Han nationality, 48.8% had a senior high school or lower level of education, and 20% lived with a monthly family income lower than 425 dollars (~ 471 dollars, considered a low family income in China). The mean time after delivery was 17.4 (± 7.2) months. Half of the participants’ waist circumference was over 80 cm, and the mean BMI was 23.56 kg/m^2^ (SD = 3.71). Demographic and clinical characteristics did not differ between intervention and control groups at baseline (*p* > 0.05). More detailed data are provided in Table 2.

**Table 2:** Baseline characteristic by groups

Variables	Total (*N* = 320)	Intervention group (*n* = 160)	Control group (*n* = 160)
Age, years	31.92 (4.91)	32.14 (5.03)	31.71 (4.79)
^b^Ethnicity			
Han ethnic group	177 (55.3%)	85 (53.1%)	92 (57.5%)
Ethinic minority (non-Han)	143 (44.7%)	75 (46.9%)	68 (42.5%)
Education			
Junior high school and below	68 (21.3%)	30 (18.8%)	38 (23.7%)
Senior high school	88 (27.5)	42 (26.2)	46 (28.8)
College and above	164 (51.2%)	88 (55.0%)	76 (47.5%)
Occupation			
Part-time job or no job	123 (38.4%)	54 (33.8%)	69 (43.1%)
Full-time job	197 (61.6%)	106 (66.2%)	91 (56.9%)
^c^Family income per month			
< 3000 RMB (425 dollars)	63 (19.7%)	30 (18.8%)	33 (20.6%)
≥ 3000 RMB (425 dollars)	257 (80.3%)	130 (81.3%)	127 (79.4%)
Months after delivery	17.62 (17.17)	17.43 (16.50)	17.84 (17.99)
T2D Risk score, points (Range 0–88)	24.72 (6.82)	24.77 (6.47)	24.68 (7.18)
FBG, mmol/L	5.13 (0.60)	5.23 (0.61)	5.04 (0.58)
OGTT-2 h, mmol/L	6.22 (1.47)	6.48 (1.44)	5.96 (1.47)
BMI, kg/m^2^	23.65 (3.57)	23.75 (3.44)	23.56 (3.71)
Waist circumference > =80 cm	160 (50%)	87 (54.1%)	73 (49.1%)
Moderate-high physical activity	250 (78.1%)	124 (77.5%)	126 (78.8%)
Perceived Stress, points	24.34 (7.18)	25.15 (6.83)	23.54 (7.45)
negative domain (Range 7–35)	11.85 (4.15)	12.26 (4.02)	11.44 (4.25)
positive domain (Range 7–35)	12.49 (4.78)	12.88 (4.80)	12.09 (4.74)
Quality of Life, points			
Physiological domain (Range 4–20)	13.19 (1.69)	13.01 (1.65)	13.37 (1.71)
Psychological domain (Range 4–20)	13.27 (1.92)	12.83 (1.81)	13.69 (1.94)
Social relationship domain (Range 4–20)	14.89 (2.04)	14.65 (2.07)	15.12 (1.98)
Environmental domain (Range 4–20)	12.49 (1.80)	12.18 (1.59)	12.79 (1.94)
Social Support, points (Range 12–66)	44.70 (5.59)	44.44 (5.62)	44.97 (5.57)
General Self-Efficacy, points (Range 10–40)	25.44 (5.87)	25.69 (5.91)	25.20 (5.83)

^a^Data are presented as n (%) or n/N (%), or mean (SD)

^b^We divided the ethnicity into two categories: Han ethnic group and Ethnic minorities (non-Han, including Tujia, Miao, Hui and other 52 minorities). Compared with Han ethnic group, ethnic minorities have their own distinctive ways of lifestyle, culture, religions, and history

^c^The average number of family members is 4.86 for the total sample, 4.71 for ILSM group, and 5.01 for control group

At the 6-month follow-up, 245 participants (76.6%) completed measurements (127 in the ILSM group and 118 in the control group); 287 participants (89.7%) completed measurements at 18-month follow-up (138 in the ILSM group and 149 in the control group). The overall attendance rate was 72% for the in-person sessions and telephone consultations of the ILSM program. The final program fidelity was 98%. There were no significant differences between participants who completed measurements and missed measurements on demographic and clinical characteristics (*p* > 0.05).

### Changes in T2D risk scores

**Fig. 2:**
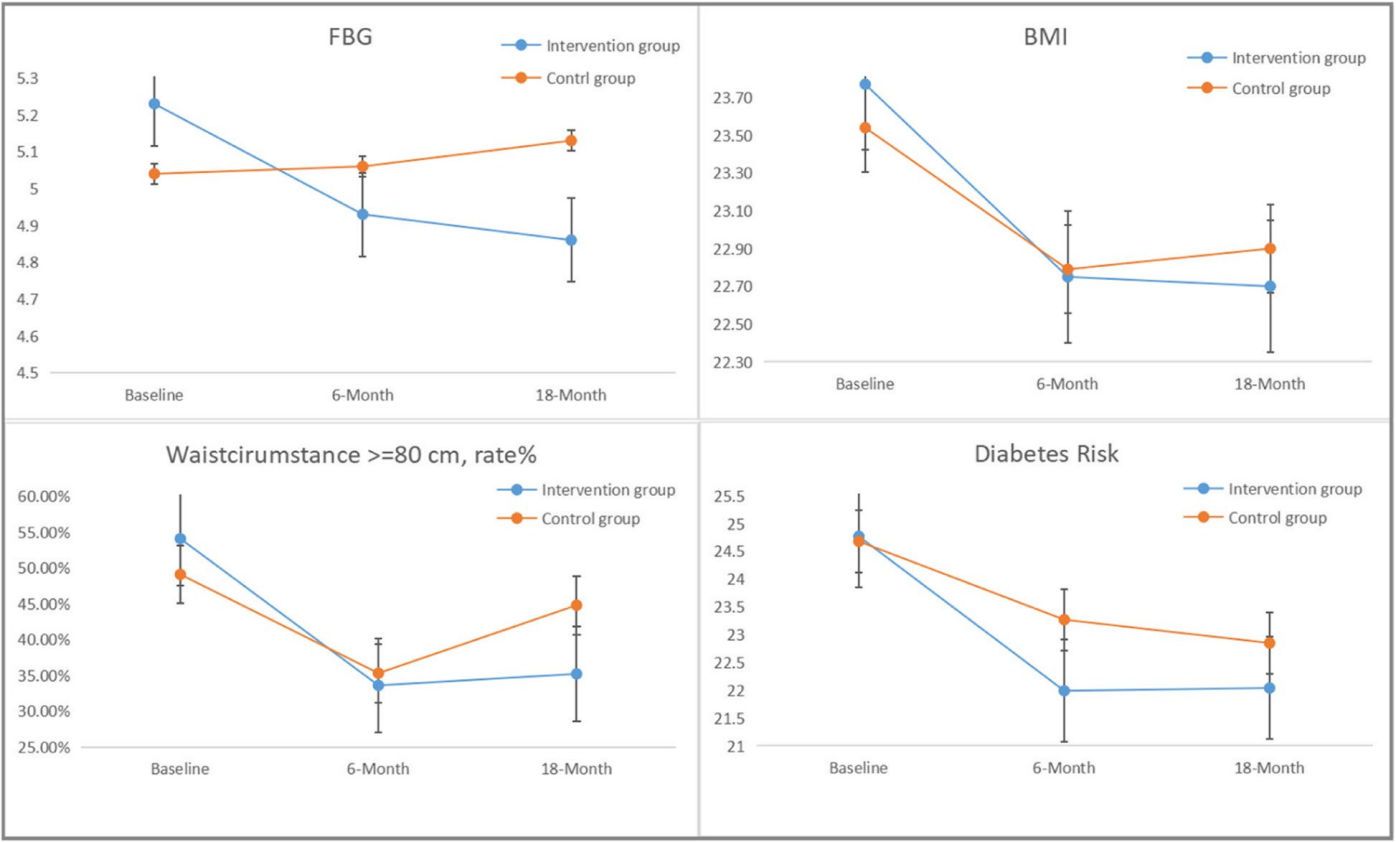
Indicated the change in FBG (fast blood glucose), BMI (body mass index), waist circumference and diabetes risk between two groups over 18-months

Taking into account the three-time points and adjusting for baseline variables and the cluster effect, there was a significant decline in T2D risk scores in the intervention group compared to the control group over time (β = − 0.411 [95%CI -0.815, − 0.008]; *p* = 0.046, adjusted) (Table 3 and Fig. 2).

**Table 3:** The 18-Month Efficacy of the Intensive Lifestyle Modification Program on T2D risk scores, Glycemic, Weight-related, Behavioral, and Psychosocial Outcomes: the GEM trial^a^

	Intervention Group (*n* = 160)	Control Group (*n* = 160)	Generalized Estimation Equation Model			
			Unadjusted Intervention Effect		Adjusted Intervention Effect	
			β (95%CI)	*p*-value	β (SE)	*p*-value
**T2D Risk Score, points**			−0.379 (−0.753 to − 0.004)	0.047	− 0.411 (− 0.815 to − 0.008)	0.046
Baseline	24.77 (6.47)	24.68 (7.18)				
6-Month	21.99 (4.65)	23.27 (6.30)				
18-Month	22.04 (5.68)	22.85 (6.13)				
**FBG, mmol/L**			−0.172 (− 0.240 to − 0.104)	<.001	− 0.169 (− 0.252 to − 0.087)	<.001
Baseline	5.23 (0.61)	5.04 (0.58)				
6-Month	4.93 (0.99)	5.06 (0.77)				
18-Month	4.86 (0.70)	5.13 (0.89)				
**OGTT-2 h, mmol/L**			−0.090 (−0.241 to 0.061)	0.241	−0.101 (− 0.293 to 0.091)	0.303
Baseline	6.48 (1.44)	5.96 (1.47)				
6-Month	6.00 (1.60)	6.12 (1.72)				
18-Month	6.38 (1.62)	6.07 (1.93)				
**BMI, kg/m**^**2**^			−0.188 (−0.367 to −0.009)	0.039	−0.194 (− 0.373 to − 0.015)	0.034
Baseline	23.75 (3.44)	23.56 (3.71)				
6-Month	22.37 (3.94)	21.83 (3.56)				
18-Month	22.70 (2.92)	22.90 (3.22)				
**Waist Circumference > =80 cm, rate%**			−0.179 (−0.357 to −0.002)	0.047	−0.182 (− 0.358 to − 0.004)	0.045
Baseline	54.10%	49.10%				
6-Month	33.60%	35.30%				
18-Month	35.20%	44.80%				
**Total physical activity level, moderate or high, rate% week**			−0.185 (−0.444 to 0.074)	0.161	−0.129 (− 0.485 to 0.227)	0.477
**rate%**						
Baseline	124 (77.5%)	126 (78.8%)				
6-Month	138 (86.3%)	135 (84.3%)				
18-Month	145 (90.6%)	137 (85.6%)				
**Intention to eat low glycemic index food**			2.714 (0.897 to 4.531)	0.003	2.879 (0.650 to 5.107)	0.011
Baseline	108.98 (20.67)	106.78 (21.15)				
6-Month	111.37 (17.60)	107.98 (20.15)				
18-Month	115.62 (17.41)	104.5 (21.16)				
**Perceived Stress**			−0.539 (−1.143 to 0.066)	0.081	−0.605 (−1.355 to 0.145)	0.114
Baseline	25.15 (6.83)	23.54 (7.45)				
6-Month	23.91 (7.16)	24.06 (6.53)				
18-Month	23.34 (6.46)	23.71 (7.23)				
Negative domain			−0.339 (−0.697 to 0.019)	0.064	−0.444 (− 0.858 to − 0.029)	0.036
Baseline	12.26 (4.02)	11.44 (4.25)				
6-Month	12.01 (4.52)	11.68 (3.64)				
18-Month	11.56 (3.95)	12.02 (3.68)				
Positive domain			−0.245 (−0.685 to 0.194)	0.274	0.128 (−0.689 to 0.433)	0.654
Baseline	12.88 (4.80)	12.09 (4.74)				
6-Month	11.90 (4.79)	12.38 (4.94)				
18-Month	11.78 (4.96)	11.68 (5.14)				
**Quality of life**						
Physiological domain			0.057 (−0.128 to 0.242)	0.548	0.165 (− 0.060 to 0.390)	0.150
Baseline	13.01 (1.65)	13.37 (1.71)				
6-Month	14.72 (1.97)	14.73 (1.82)				
18-Month	14.70 (1.98)	14.97 (1.89)				
Psychological domain			0.380 (0.177 to 0.583)	<.001	0.464 (0.219 to 0.709)	<.001
Baseline	12.83 (1.81)	13.69 (1.94)				
6-Month	14.24 (2.13)	14.23 (2.03)				
18-Month	14.55 (2.20)	14.28 (2.10)				
Social relationship domain			0.113 (−0.078 to 0.304)	0.245	0.151 (−0.083 to 0.385)	0.206
Baseline	14.65 (2.07)	15.12 (1.98)				
6-Month	15.09 (2.46)	15.27 (1.93)				
18-Month	15.14 (2.13)	15.33 (1.71)				
Environmental domain			0.293 (0.110 to 0.476)	0.002	0.335 (0.116 to 0.554)	0.003
Baseline	12.18 (1.59)	12.79 (1.94)				
6-Month	14.03 (2.11)	13.77 (2.05)				
18-Month	14.43 (2.23)	14.24 (1.93)				
**Social Support**			0.371 (−0.121 to 0.864)	0.139	0.747 (0.131 to 1.364)	0.018
Baseline	44.44 (5.62)	44.97 (5.57)				
6-Month	44.13 (6.41)	44.23 (6.47)				
18-Month	45.99 (6.08)	45.29 (6.00)				
**General Self-Efficacy**			−0.250 (−0.793 to 0.293)	0.367	−0.113 (− 0.784 to 0.558)	0.742
Baseline	25.69 (5.91)	25.20 (5.83)				
6-Month	24.64 (5.03)	24.89 (5.00)				
18-Month	25.42 (5.89)	25.71 (5.35)				

^a^Models were adjusted for age, months after delivery, ethnicity, education (two levels: Senior high school and below and College and above) marriage, occupation (two levels: part-time job or no job, and full-time job), family income (two levels:≤3000 RMB and > 3000RMB per month), and cluster

### Changes in glycemic and weight-related outcomes

Participants in the intervention group experienced a significant decline of 0.37 mmol/L in FBG compared with 0.09 mmol/L in the control group from baseline to 18-month follow-up (β = −.169 [95%CI −.252, −.087]; *p*<.001, adjusted). There were no significant differences in OGTT-2 h between groups over time, yet OGTT-2 h had a 0.1 mmol/L decline in the intervention group with an increase of 1.11 mmol/L in the control group (β = .034 [95%CI −.081, −.031]; *p* = .157, adjusted).

There was a significant decrease of 1.07 kg/m^2^ in BMI in the intervention group compared with a decrease of 0.64 kg/m^2^ in the control group (β = −.194 [95%CI −.373, −.015]; *p* = 0.034, adjusted) at 18-month follow up. The percentage of participants with a waist circumference over 80 cm also significantly declined (β = −.182 [95%CI −.358, −.004]; *p* = .045, adjusted) in the intervention group compared to the control group (18.90% in intervention group vs. 4.30% in control group) (Table 3 and Fig. 2).

### Changes in behavioral outcomes

We found a significant improvement of the intention to eat low-glycemic index food in the intervention group, whereas it declined in the control group over 18 months (+ 6.64 points vs. -2.28 points; β = 2.879 [95%CI −.776, .224]; *p* = 0.003, adjusted) (Table 3). However, no significant differences were observed in moderate and high physical activity levels (β = − 0.129 [95%CI -0.485, 0.227]; *p* = .477, adjusted).

### Changes in psychosocial outcomes

The intervention group participants reported a significant decrease in perceived negative stress, while the control group reported an increase in perceived negative stress over time (β = −.444 [95%CI −.858, −.029]; *p* = 0.036, adjusted). A significant improvement in quality of life in psychological and environmental domains was demonstrated in the intervention group compared to the control group over time (β = .464 [95%CI .219, .709]; *p*<.001, adjusted; β = .335 [95%CI .116, .554]; *p* = .003, adjusted, respectively). The intervention group also reported significantly more social support compared to the control group over time (β = .747, [95%CI .131, 1.364]; *p* = .018, adjusted). (Table 3).

### Changes in T2D risk scores by subgroups

**Fig. 3:**
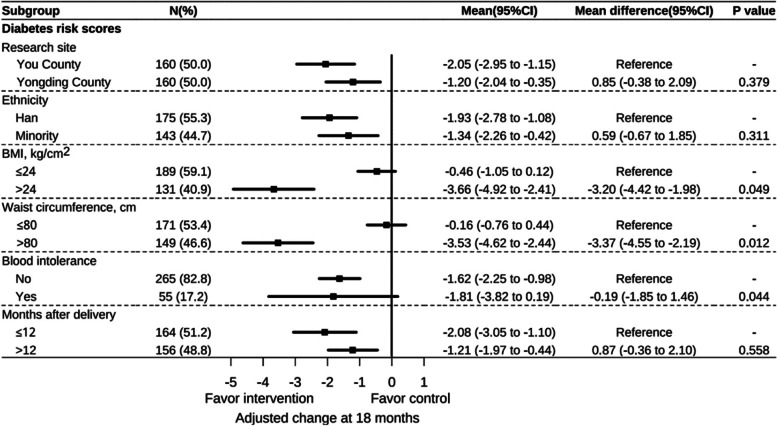
Forest plot of intervention effect at 18 months on T2D risk scores and BMI by subgroup. Interaction between intervention group and subgroup

In the subgroup analysis, the intervention was more effective for women with BMI > 24 kg/ m2 (95% CI − 4.42 to − 1.98, *p* = .049), waist circumference > 80 cm (95% CI, − 4.55 to − 2.19, *p* = .012) and blood glucose intolerance (95% CI, − 1.85 to 1.46, *p* = .044) compared to those who had relatively normal BMI, waist circumference and blood glucose at 18 months. Figure 3 displays the results of the subgroup analyses in T2D risk scores and BMI at 18-month.

## Discussion

In this study, we demonstrated that ILSM significantly reduced T2D risk scores and obtained statistically significant benefits for FBG, BMI, and intention to eat low-index glycemic food at an 18-month follow-up based on the intention-to-treat analysis. This is encouraging as previous studies reported that the benefit of lifestyle interventions for diabetes prevention was typically sustained at 6 to 12 months [7]. This exciting result may suggest that early behavior interventions are expected to touch off a series of reciprocally reinforcing recursions and interactions via goal setting and ongoing environmental support, eventually driving intervention influence over time. Also, we found that women who were overweight, had abdominal adiposity, or had blood glucose intolerance at baseline benefited more from the ILSM, thus identifying the targeted population for future implementation. In addition, this program indicated the potential of local nurses in providing lifestyle and preventive interventions in primary care centers. Overall, the tailored lifestyle intervention program was demonstrated to be effective in diabetes prevention for women with prior GDM in a low-resource rural setting. The model of care may be considered in other settings and counties.

Consistent with our result in the six-month follow-up, the intervention effect on reducing T2D risk scores was sustained at 18-month [21]. The longer-term maintenance of reduction in T2D risk score could be attributed to the lasting improvement of these modifiable T2D risk factors, such as behavioral and psychosocial factors. Our results are similar to prior studies that support the mediating effect of improvement of health behaviors and psychosocial variables on T2D risk reduction [39]. Subsequent decreases in BMI and waist circumference which were classified as modifiable T2D risk factors also decreased T2D risk scores.

Improvement of glucose markers has been identified as the key indicator of delaying progression to T2D among people with dysglycemia [40]. In this study, we observed significant improvement for FBG at 18-month, which was also found in our 6-month result. The long-lasting effect of ILSM may be explained by sustained lifestyle changes of rural women with prior GDM after intervention [41]. However, the long-term glycemic benefits obtained in ILSM was inconsistent with the finding of a recent meta-analysis, in which no benefit was found regarding measures of glycemia [42]. The plausible explanation is included interventions lacked explicit theoretical frameworks of behavior change maintenance, which could provide guidance on the development and evaluation of interventions promoting sustained change in health behaviors [43, 44]. In terms of OGTT-2 h, the significant difference between two groups was not detected at 18-month follow up, though it was improved at 6-months. The differential efficacy may be due to limited improvement room for OGTT-2 h [21], as the majority of women with GDM returned to normal glycemic levels without specific intervention in the year after delivery based on current evidence [45]. The early return to normal glycemic levels could potentially reduce the adverse effect of high glycemic levels on women’s health [46].

Over 18 months, a significant reduction in BMI and waist circumference were determined in the intervention group compared with the control group. However, consistent with most previous studies, the maximum reduction was achieved at 6-month with a plateau occurred after 6 months [47, 48]. According to the American Diabetes Association’s Adult Weight Management Evidence-Based Nutrition Practice Guideline, interventions for weight loss should last at least 6 months with a weight-maintenance program [47]. In our study, we provided three-month intensive core intervention as well as three monthly phone calls as the maintenance component for participants, in order to prevent weight regain without ongoing support [49]. As a result, the maintenance of BMI and waist circumference was demonstrated in the ILSM group, while these weight-related outcomes returned to baseline in the control group.

At 18-month, we found that women in the ILSM group have increased intentions to eat low glycemic-index food (e.g., dark green leafy vegetables, fish and seafood, whole grains or mixed) compared with the control group, although this difference between groups was not demonstrated at 6-months [50]. This may be because the adaptation of new eating habits is a complex and gradual process, and the increased intention to eat low glycemic-index food intake may have taken longer. In the dietary sessions of the ILSM, the optimization of carbohydrate composition was also included in the ILSM intervention by recommending low glycemic-index food to avoid fluctuations in post prandial blood glucose levels [51].

The difference in physical activity was not statistically significant between the two groups over 18 months. Similar results were also reported in other lifestyle interventions for women with a history of GDM. According to literature, barriers to exercise among this population include competing demands for childcare, career pressure and family responsibility as well as an established ethic of care that prioritizes the needs of others (e.g., family and friends) above their own [6, 42], although they believe that physical activity is important in the prevention of T2DM. It also suggests additional strategies, such as activity monitoring or group support, may be needed to increase physical activity in this population.

We found significant improvement in social support and perceived stress over 18 months, which is consistent with other studies [6, 52]. The ILSM program included sessions on family support and stress coping. The importance of involving all family members in diabetes prevention efforts was emphasized and a wide range of psychological strategies were provided, in order to develop sustainable healthy lifestyles with the help of their family [53] and help participants magnify their positive experiences and contribute to women’ confidence in coping with stressful situations [23, 54]. Consequently, the ILSM group participants obtained more psychological resources; thus, their QoL in the psychological domain also improved at longer-term follow-up.

The results of our subgroup analyses suggest that women with glycemic intolerance and overweight or abdominal obese women are more sensitive to the ILSM than women with relatively normal blood glucose, weight, and waist circumference in reducing T2D risk score. As glycemic markers and weight-related outcomes are important predictive indicators for future T2D risk, and women with glycemic intolerance or weight issues have a higher risk for T2DM. This intervention is particularly helpful in reducing their risk. Thus, we can reasonably infer that higher-risk groups may benefit more from lifestyle intervention programs as their physical health is in jeopardy, and they have more room for improvement in terms of glycemic level and weight [55].

This study is one of the few intervention studies with a long-term follow-up. This study has several strengths. First, we conducted a multi-dimensional evaluation of an evidence-based intervention, adapted to the local context, and adhered to the theoretical framework of behavior maintenance. Second, we designed a contextually-tailored lifestyle intervention, which was implemented effectively by local nurses with few resources; thus, it provides the possibility to improve primary healthcare in impoverished health system. More importantly, the ILSM may be used as a model for designing health-related interventions at underserved public health settings. Lastly, a robust RCT study design, including cluster randomization, excellent protocol implementation, and well-validated questionnaires are study strengths.

There were several study limitations. Due to budget and energy constraints, the sample size was calculated based on the power of our primary outcome; thus, we may not have enough power to detect meaningful, significant differences in all outcomes. Second, the improvement of the ILSM group may be due to the fact that they were given more attention (six biweekly group seminars and eight telephone consultation sessions) than the control group who did not have the same time engagement. Thirdly, the self-report measurements for physical activity may have recall bias. Lastly, the findings of subgroup analysis should be considered exploratory and should not be over-interpreted given the number of comparisons reported. Future researches are needed to confirm our findings.

Despite these limitations, our findings have several implications. First, it is important to modify lifestyle interventions to the context of women’s lives and to address their barriers to long-term lifestyle change. For example, maintenance interventions via smartphones may be feasible in this population; and strategies that promote self-directed behavior change could be incorporated [56]. In addition, studies with larger sample sizes to detect changes in behavioral or other outcomes are needed. Lastly, the ILSM program has the potential to be served as an effective model of diabetes prevention for high-risk groups in underserved settings.

## Conclusion

In conclusion, the ILSM provided robust evidence to support lifestyle interventions in preventing T2D wasn’t weakened by time, but can preserve and strengten it over 18 months in women with a history of GDM in low-resource rural settings in China. Women with a history of GDM who are currently overweight, have abdominal adiposity, or have blood glucose intolerance may benefit more from a lifestyle program and should be the targeted population for further dissemination and implementation. This program is a promising model of diabetes prevention, reducing health disparities in low-resource settings about diabetes prevention globally.

## Supplementary Information

**Additional file 1: Appendix I.** Fidelity Checklist of Intensive LifeStyle Modification Program.

## Data Availability

The datasets used during the current study are available from the corresponding author on reasonable request.

## Abbreviations

BMI: Body mass index
CHINARISK: Chinese Diabetes Risk Scale
FBG: Fasting blood glucose
GDM: Gestational diabetes mellitus
GEE: Generalized estimation equation
ILSM: Intensive LifeStyle Modification Program
OGTT: Oral glucose tolerance test
RCT: Randomized controlled trial
T2D: Type 2 diabetes
