# Supplementary material for: The 18-month efficacy of an Intensive LifeStyle Modification Program (ILSM) to reduce type 2 diabetes risk among rural women: a cluster randomized controlled trial
Source: Global Health. 2023 Jan 26;19:6. doi: 10.1186/s12992-023-00910-3 (PMC9881320; doi:10.1186/s12992-023-00910-3)
Supplement: Supplementary file 1 — Additional file 1: Appendix I. Fidelity Checklist of Intensive LifeStyle Modification Program. [file 12992_2023_910_MOESM1_ESM.docx]

**Appendix I:**

**Fidelity Checklist of** **Intensive LifeStyle Modification Program**

**Kindly Note:** The fidelity checklist is developed and validated by the Prevention Research Center, at Colorado State University^1^. It is not specific to this program but could be used by observers in general.

| Facilitator Name(s): | |
| --- | --- |
| Observer Name(s): | |
| Date of Session: | |
| Session Number:  Session Title:  Session Start Time:  Session End Time: | Session Location:  Total Number of Participants:  Number of participants arriving late:  Number of participants leaving early: |

| **Section 1: Adherence**  The extent to which core components of a program are utilized (i.e., using original instructional techniques and procedures) | | | | | | |
| --- | --- | --- | --- | --- | --- | --- |
| 1 | During this session, program materials (including videos, readings, posters, slideshows, etc.) were delivered as specified in the program manual and session plan. | 1-Strongly  Disagree | | 2-  Disagree | 3-  Agree | 4-  Strongly  Agree |
| 2 | During this session, how often were any program activities (including worksheets, handouts, videos, games,  etc.) omitted or removed? | 1-Strongly  Disagree | | 2-Disagree | 3-Agree | 4-  Strongly  Agree |
| 3 | During this session, were any program activities (including worksheets, handouts, videos, games, etc.) replaced with an alternative activity? | 1-Strongly  Disagree | | 2-Disagree | 3-Agree | 4-  Strongly  Agree |
| 4 | During this session, were any activities or resources added to the program as supplemental/additional materials? | 1-Strongly  Disagree | | 2-Disagree | 3-Agree | 4-  Strongly  Agree |
| **Notes:** | | | | | | |
| **Total Points =** | | | **Maximum Points = 16** | | | |

| **Section 2: Exposure**  i.e., dosage—number, frequency, and length of program sessions | | | | | | |
| --- | --- | --- | --- | --- | --- | --- |
| 1 | Participant attendance was logged for this session. | 1-Strongly  Disagree | | 2-  Disagree | 3-  Agree | 4-  Strongly  Agree |
| 2 | The activities in this session were delivered in the sequence intended by the program developers. | 1-Strongly  Disagree | | 2-Disagree | 3-Agree | 4-  Strongly  Agree |
| 3 | The delivery of this session took the amount of time as intended by the program developers. (Note that this is not the same as the time intended by your organization.)  →*Provide notes in the section below about how the surplus or shortage of time was compensated.* | 1-Strongly  Disagree | | 2-Disagree | 3-Agree | 4-  Strongly  Agree |
| **Notes:** | | | | | | |
| **Total Points =** | | | **Maximum Points = 12** | | | |

| **Section 3: Quality of Delivery**  The quality and thoroughness in preparation, skills, leadership, and enthusiasm | | | | | | |
| --- | --- | --- | --- | --- | --- | --- |
| 1 | During this session, all supplies/ materials that are indicated in the program manual were available. | 1-Strongly  Disagree | | 2-  Disagree | 3-  Agree | 4-  Strongly  Agree |
| 2 | During this session, the amount of physical space was adequate for this session (For virtual settings refer to  internet accessibility). | 1-Strongly  Disagree | | 2-Disagree | 3-Agree | 4-  Strongly  Agree |
| 3 | During this session, the quality of space was adequate for this session (For virtual settings refer to the quality of virtual space). | 1-Strongly  Disagree | | 2-Disagree | 3-Agree | 4-  Strongly  Agree |
| 4 | During this session, the facilitator(s) appeared to be prepared with the skills/knowledge to facilitate this  Session. | 1-Strongly  Disagree | | 2-Disagree | 3-Agree | 4-  Strongly  Agree |
| 5 | During this session, the facilitator(s) demonstrated enthusiasm and topic interest during this session. | 1-Strongly  Disagree | | 2-Disagree | 3-Agree | 4-  Strongly  Agree |
| 6 | During this session, the facilitator(s) demonstrated positive leadership and a positive attitude. | 1-Strongly  Disagree | | 2-  Disagree | 3-  Agree | 4-  Strongly  Agree |
| 7 | During this session, the facilitator(s) provided a welcoming greeting when the participants entered the room. | 1-Strongly  Disagree | | 2-Disagree | 3-Agree | 4-  Strongly  Agree |
| 8 | During this session, the facilitator(s) clearly explained topics and activities. | 1-Strongly  Disagree | | 2-Disagree | 3-Agree | 4-  Strongly  Agree |
| 9 | During this session, the facilitator(s) appeared to feel comfortable interacting with the participants. | 1-Strongly  Disagree | | 2-Disagree | 3-Agree | 4-  Strongly  Agree |
| 10 | During this session, you are confident that the facilitator(s) provided high-quality facilitation of this session. | 1-Strongly  Disagree | | 2-Disagree | 3-Agree | 4-  Strongly  Agree |
| **Notes:** | | | | | | |
| **Total Points =** | | | **Maximum Points = 40** | | | |

| **Section 4: Participant Responsiveness**  The participants' engagement with the program | | | | | | |
| --- | --- | --- | --- | --- | --- | --- |
| 1 | During this session, participants were interested in the material. | 1-Strongly  Disagree | | 2-  Disagree | 3-  Agree | 4-  Strongly  Agree |
| 2 | During this session, participants were engaged in the material. | 1-Strongly  Disagree | | 2-Disagree | 3-Agree | 4-  Strongly  Agree |
| 3 | During this session, members were participating in the discussions/ activities. | 1-Strongly  Disagree | | 2-Disagree | 3-Agree | 4-  Strongly  Agree |
| 4 | During this session, participants  understood/comprehended the material.  →*Elaborate & provide examples in the notes below.* | 1-Strongly  Disagree | | 2-Disagree | 3-Agree | 4-  Strongly  Agree |
| **Notes:** | | | | | | |
| **Total Points =** | | | **Maximum Points = 16** | | | |

**Final Notes on Special Circumstances:**

| In addition to the notes above, please describe any unusual circumstances that arose (such as disturbances) that might have affected the group's dynamics for this session: |
| --- |

**Overall Fidelity* =**

*** Add the total points of all 4 sections together and divide by the maximum points.**

[1] Prevention Research Center, Colorado State University. (n.d.). Fidelity checklists designed by the PRC for General Use (Observer report). *The Website of Prevention Research Center, Colorado State University*. Retrieved June 16, 2022, from [Fidelity Checklists - Prevention Research Center (colostate.edu)](https://www.chhs.colostate.edu/prc/implementation-toolbox/ebp-fidelity-checklists-logic-models/fidelity-checklists-for-select-evidence-based-programs/).
